# Sparse Bayesian classification and feature selection for biological expression data with high correlations

**DOI:** 10.1371/journal.pone.0189541

**Published:** 2017-12-27

**Authors:** Xian Yang, Wei Pan, Yike Guo

**Affiliations:** 1 Data Science Institute, Imperial College London, London, SW7 2AZ, United Kingdom; 2 Department of Cognitive Robotics, Delft University of Technology, Delft, Netherlands; Harbin Institute of Technology Shenzhen Graduate School, CHINA

## Abstract

Classification models built on biological expression data are increasingly used to predict distinct disease subtypes. Selected features that separate sample groups can be the candidates of biomarkers, helping us to discover biological functions/pathways. However, three challenges are associated with building a robust classification and feature selection model: 1) the number of significant biomarkers is much smaller than that of measured features for which the search will be exhaustive; 2) current biological expression data are big in both sample size and feature size which will worsen the scalability of any search algorithms; and 3) expression profiles of certain features are typically highly correlated which may prevent to distinguish the predominant features. Unfortunately, most of the existing algorithms are partially addressing part of these challenges but not as a whole. In this paper, we propose a unified framework to address the above challenges. The classification and feature selection problem is first formulated as a nonconvex optimisation problem. Then the problem is relaxed and solved iteratively by a sequence of convex optimisation procedures which can be distributed computed and therefore allows the efficient implementation on advanced infrastructures. To illustrate the competence of our method over others, we first analyse a randomly generated simulation dataset under various conditions. We then analyse a real gene expression dataset on embryonal tumour. Further downstream analysis, such as functional annotation and pathway analysis, are performed on the selected features which elucidate several biological findings.

## Introduction

Biological system is being comprehensively profiled by various expression data through high-throughput technologies, such as gene expression data (measured by the microarray or next generation sequencing technology), protein expression expression (measured by the mass spectrometry-based flow cytometer) and medical imaging (measured by functional magnetic resonance imaging or computerised tomography scan) [1, 2]. Computational and statistical methods for discovering functional roles of features from expression data are required to have the ability of handling large scale datasets. A straightforward analysis is to carry out statistical tests to identify differentially expressed features between groups of samples [3]. Functional analyses, such as the Gene Set Enrichment Analysis (GSEA) [4], can be followed to discover pathways or biological functions that are over-expressed in the differential feature list. Then the biological semantics of differential features can be explored. Besides differential feature discovery, another important type of analysis is sample classification, in which case patient samples are classified by characteristics such as disease subtypes and treatment strategies [5]. The classification model constructed from biological expression data can be used for disease diagnosis [6, 7] or clinical outcome prediction [8, 9]. In this paper, we focus on investigating classification methods to build predictive models from biological expression data.

There are a large range of machine learning methods to construct classification models. Examples of such methods include deep learning [10, 11], graphical models [12, 13], regularised Bayesian models [14], nonparametric Bayesian models [15, 16] and also some traditional methods such as support vector machine (SVM) [17], linear discriminant analysis [18] and Gaussian Naive Bayes [19]. Many tools are particularly designed for biological data. For example, a Python package called Pse-Analysis [20], is developed to automatically generate classifiers for genomics and proteomics datasets. It is based on the framework of LIBSVM [21] and inherits the characteristics of the SVM method. Pse-Analysis carries out the following five key tasks: feature extraction, parameter selection, model training, cross validation and evaluation. Another example is the iDHS-EL method mainly designed for DNA sequence data [22]. iDHS-EL uses three different ways to extract feature vector to represent sequence data, which leads to three different basic random forest (RF) predictors. Then the ensemble predictor is formed by using these three predictors. RF is a combination of decision trees, which has the ability to select important features.

Among various classification methods, sparse Bayesian Learning (SBL) [23, 24] is featured in overcoming the dimensionality problem. This is a common issue of applying classification methods to large scale data: the number of features is usually several orders of magnitudes over the samples. In our application, feature size (number of measured features) is much larger than sample size. In this case, many classification methods cannot work as well as SBL. For example, authors in [25, 26] find that SBL outperforms decision tree methods. SBL only uses a small subset of input features for prediction, based on the observation that relevant features are sparse compared to the dimension of whole feature space. Bayesian inference is adopted to obtain solutions for probabilistic classification. SBL is in the same functional form of SVM, but provides probabilistic classification. Unlike SVM, benefited from Bayesian formulation, SBL avoids the setting of free parameters which requires the cross-validation process. More specifically, SBL uses a fully probabilistic framework and introduces a prior over the model weights governed by a set of hyperparameters. Each hyperparameter is associated with each weight, whose most probable value is iteratively estimated from the data through an expectation-maximisation (EM) procedure. Sparsity is achieved by finding posterior distributions of many weights are sharply peaked at zero.

One of the distinguishing characteristics of SBL is that it does not only build a classification model but also returns a set of features with non-zero weights. In our application, these features are predictive molecules, differentiating two groups of samples. Thus, we can also regard the SBL as a feature selection method. Traditional feature selection methods include statistics tests to reduce feature space by examining whether the significant values of features of a test pass the predefined threshold. For biological data, there are many advanced feature selection methods being proposed. For example, the binary particle swarm optimisation (BPSO) based model is proposed in [27] for the gene selection of Microarray data. To improve the performance of feature selection, BPSO uses gene-to-class sensitivity (GCS) information in the feature selection process. GCS information is obtained from gene expression data indicating whether a gene is sensitive to sample classes. To evaluate candidate gene subsets selected from BPSO, extreme learning machine (ELM) is used for classification model construction. Unlike this method, SBL, similar to other embedded feature selection methods, integrates the feature selection step into the predictive model construction.

There are some examples of embedded feature selection methods which achieve the feature selection by imposing regularisation on existing classification methods, such as regularised SVM [28, 29] and sparse logistic regression [30, 31]. These methods need to tune the regularization parameters via the cross validation process. The work in [32] develops a Bayesian approach based on a probit regression model with a generalised singular *g*-prior distribution for regression coefficients. The hyperparameters need to be predefined in the model and the selected feature set is quite small. With limited number of selected features, it is not easy to discover biological functions. The main reason that only a quite small feature set is selected is correlated features are not selected coherently. As we know that in a biological process, multiple molecules are working together, resulting in correlated feature expression levels. SBL, imposed with sparsity constraints, cannot simultaneously select correlated features. Instead, one out of a set of correlated features is usually selected in the predictive model. In the literature, several approaches have been proposed for classification and feature selection and some of them are actually based on SBL [33–35]. Not surprisingly, the feature set generated by the Bayesian selection method in [35] is quite small that only 12 out of 7128 genes in an example gene expression dataset are selected. The feature set returned from these methods cannot be easily used to discover predictive pathways or biological functions.

Motivated by the fact that existing classification methods either fail to identify a list of predictive features or easily discard correlated features, we propose a computational method derived from SBL to simultaneously build a classifier and select predictive features which are highly correlated. Our classification model is constructed through an iterative convex optimisation procedure instead of a one-step closed form calculation. Moreover, the optimisation-centric formulation of our method can be easily paralleled [36]. The cost function is cast using hierarchical Bayesian model, where the parameters’ prior distribution is parameterised by the hyperparameter. Its main goal is to infer the posterior distribution of parameter via Bayes’ rule. Rather than using the EM procedure to update parameters and hyperparameters, our method infer both parameters and hyperparameters via the convex optimisation procedure [24, 37]. The paper is organised as follows, we first detailed the method and then test against simulated and real datasets. In the simulation study, we compare the performance of our method with other methods in the aspects of classification and feature selection abilities. In the real dataset analysis, we apply our method to construct a classification model to predict different types of tumours. The selected features in the predictive model are fed into downstream analyses for biological functions and pathways discovery. The results show that our method can perform classification and feature selection at the same time, while the selected features can give insight into new functional modules.

## Methods

The proposed classification method constructs a mathematical model through an iterative optimisation procedure. The resulting model can simultaneously perform classification and return a relevant feature set. In this section, we first follow the sparse Bayesian approach to define a single target optimisation function to obtain both parameters and hyperparameters. Then we infer the equations to iteratively solve the optimisation problem via the smooth-concave procedure. The standard EM updating procedure is replaced by the optimisation procedure.

### Optimisation problem definition

Suppose we get a set of input vectors ${\left\{x_{n}\right\}}_{n=1}^{N}$ along with corresponding targets ${\left\{y_{n}\right\}}_{n=1}^{N}$. We wish to learn the underlying functional mapping which is defined by a parameterised function $f\left(x;\beta \right)=\sum_{i=1}^{M}\beta_{i}\phi_{i}\left(x\right)$, where the output is the linear weighted sum of *M* basis functions and ***β*** = [*β*_1_, *β*_2_, …, *β*_*M*_]^⊤^ contains the parameter. Let **Φ** be the *N* × *M* design matrix with **Φ** = [***ϕ***(**x**_1_), ***ϕ***(**x**_2_), …, ***ϕ***(**x**_*N*_)]^⊤^, wherein ***ϕ***(**x**_*n*_) = [*ϕ*_1_(**x**_*n*_), *ϕ*_2_(**x**_*n*_), …, *ϕ*_*M*_(**x**_*n*_)]. Then we can express the mapping function as *f*(**x**; ***β***) = **Φ*β***. Usually, the prior distribution of the weights is assumed to follow a zero-mean isotropic Gaussian:

$$\begin{array}{c} P\left(\beta |\gamma \right)=\prod_{i=1}^{M}N\left(\beta_{i}|0,\gamma_{i}\right)=\prod_{i=1}^{M}{\left(2\pi \gamma_{i}\right)}^{-\frac{1}{2}}\exp\left\{-\frac{\beta_{i}^{2}}{2\gamma_{i}}\right\}, \end{array}$$ (1)

where

$$\gamma =[\gamma_{1},\ldots ,\gamma_{M}]\in R^{M},\;\Gamma =\mathrm{diag}[\gamma ].$$ (2)

Here, we focus on investigating the case that the target variable is binary. The likelihood function P(y|β,x) is expressed in the form of the logistic regression model:

$$\begin{array}{c} \log P\left(y|\beta ,x\right)=-\sum_{n=1}^{N}\log\left[1+\exp\left\{-y_{n}\beta^{⊤}\phi \left(x_{n}\right)\right\}\right]. \end{array}$$ (3)

According to the Bayes’ rule, the posterior distribution over weights P(β|y,γ) is proportional to P(y|β,x)P(β|γ). Maximisation of posterior is equivalent to finding the maximum over ***β*** of

$$\begin{array}{c} \log P\left(y|\beta ,x\right)P\left(\beta |\gamma \right)=-\sum_{n=1}^{N}\log\left[1+\exp\left\{-y_{n}\beta^{⊤}\phi \left(x_{n}\right)\right\}\right]-\frac{1}{2}\beta^{⊤}\Gamma^{-1}\beta -\frac{1}{2}\log\left|\Gamma \right|. \end{array}$$ (4)

Thus, the weights can be found through

$$\begin{array}{c} \underset{\beta }{\mathrm{argmin}}E\left(\beta \right)=\underset{\beta }{\mathrm{argmin}}\sum_{n=1}^{N}\log\left[1+\exp\left\{-y_{n}\beta^{⊤}\phi \left(x_{n}\right)\right\}\right]+\frac{1}{2}\beta^{⊤}\Gamma^{-1}\beta +\frac{1}{2}\log\left|\Gamma \right|. \end{array}$$ (5)

The gradient and Hessian matrices at arbitrary point of β* are defined as

$$\begin{array}{c} g\left(\beta^{*}\right)≜\nabla E\left(\beta \right){|}_{\beta^{*}}=-\sum_{n=1}^{N}y_{n}\phi \left(x_{n}\right)\left[1-S\left\{y_{n}{\beta^{*}}^{⊤}\phi \left(x_{n}\right)\right\}\right]+\Gamma^{-1}\beta^{*} \end{array}$$ (6)

and

$$\begin{array}{c} H\left(\beta^{*}\right)≜\nabla \nabla E\left(\beta \right){|}_{\beta^{*}}=\Phi^{⊤}\mathrm{diag}[y]Z\left(\beta^{*}\right)\mathrm{diag}[y]\Phi +\Gamma^{-1} \end{array}$$ (7)

where

$$\begin{array}{c} Z\left(\beta^{*}\right)=\mathrm{diag}(\left\{S\left\{y_{n}{\beta^{*}}^{⊤}\phi \left(x_{n}\right)\right\}{\left[1-S\left\{y_{n}{\beta^{*}}^{⊤}\phi \left(x_{n}\right)\right]\right\}}_{n=1}^{N}\right) \end{array}$$ (8)

and

$$\begin{array}{c} S\left\{f\right\}=\frac{1}{\left(1+e^{-f}\right)}. \end{array}$$ (9)

The hyperparameter ***γ*** is updated by maximising the marginal likelihood, which is equivalent to

$$\begin{array}{c} \underset{\gamma }{\mathrm{argmin}}-P\left(y|\gamma ,x\right)=\underset{\gamma }{\mathrm{argmin}}-\int P\left(y|\beta ,x\right)P\left(\beta |\gamma \right)d\beta . \end{array}$$ (10)

According to Taylor expansion, we can get the following approximation at mode β*:

$$\begin{array}{c} -\log P\left(y|\beta ,x\right)P\left(\beta |\gamma \right)=E\left(\beta^{*}\right)+\frac{1}{2}{\left(\beta -\beta^{*}\right)}^{⊤}H\left(\beta^{*}\right)\left(\beta -\beta^{*}\right). \end{array}$$ (11)

Therefore, the logarithm of negative marginal likelihood is

$$\begin{array}{c} -\log P\left(y|\gamma ,x\right)=E\left(\beta^{*}\right)+\frac{M}{2}\log2\pi +\frac{1}{2}\log\left|H\left(\beta^{*}\right)\right|. \end{array}$$ (12)

Thus ***γ*** can be estimated by

$$\begin{array}{c} \underset{\gamma }{\mathrm{argmin}}E\left(\beta^{*}\right)+\frac{1}{2}\log\left|H\left(\beta^{*}\right)\right|. \end{array}$$ (13)

From Eqs (5) and (13), we can jointly estimate ***β*** and ***γ*** through a common objective function:

$$\begin{array}{c} \underset{\beta ,\gamma }{\mathrm{argmin}}\sum_{n=1}^{N}\log\left[1+\exp\left\{-y_{n}\beta^{⊤}\phi \left(x_{n}\right)\right\}\right]+\beta^{⊤}\Gamma^{-1}\beta +\log|\Gamma |+\frac{1}{2}\log\left|H\left(\beta^{*}\right)\right|, \end{array}$$ (14)

where |**H**(β*)| is the Hessian matrix calculated at mode β*, which is assumed to be obtained through the minimisation step of ***β*** in the iterative optimisation process. The reason we label * in |**H**(β*)| is to emphasize that the term |**H**| is not involved when updating ***β*** according to Eq (5).

### Iterative optimisation algorithm

The objective function defined in Eq (14) can be formulated as a convex-concave procedure (CCCP) for updating ***β*** and ***γ***, which is

$$\begin{array}{c} \underset{\beta ,\gamma }{\mathrm{argmin}}u\left(\beta ,\gamma \right)+v\left(\gamma \right), \end{array}$$ (15)

where the data fitting term *u*(***β***, ***γ***) is a smooth function in the form of

$$\begin{array}{c} u\left(\beta ,\gamma \right)≜\sum_{n=1}^{N}\log\left[1+\exp\left\{-y_{n}\beta^{⊤}\phi \left(x_{n}\right)\right\}\right]+\beta^{⊤}\Gamma^{-1}\beta , \end{array}$$ (16)

and the regularisation term *v*(***γ***) is a concave function [24, 38]:

$$\begin{array}{c} v\left(\gamma \right)=\log|\Gamma |+\log|H\left(\beta^{*}\right)|. \end{array}$$ (17)

By expressing the objective function in the convex-concave form [39], we can evoke standard iterative optimisation procedure to get its solution at the *k* + 1^th^ iteration as follows:

$$\begin{array}{c} \beta^{k+1}=\underset{\beta }{\mathrm{argmin}}u\left(\beta ,\gamma^{k}\right) \end{array}$$ (18)

and

$$\begin{array}{c} \gamma^{k+1}=\underset{\gamma }{\mathrm{argmin}}u\left(\beta^{k+1},\gamma \right)+\nabla_{\gamma }v\left(\gamma ,H\left(\beta^{k+1}\right)\right){|}_{\gamma =\gamma^{k}}^{⊤}\cdot \gamma . \end{array}$$ (19)

If we define

$$\begin{array}{c} w_{i}^{k}≜\frac{1}{\sqrt{\gamma_{i}^{k}}}, \end{array}$$ (20)

***β***^*k*+1^ can be obtained as the following expression in the form of reweighted *ℓ*_2_ regularisation:

$$\begin{array}{c} \beta^{k+1}=\underset{\beta }{\mathrm{argmin}}\sum_{n=1}^{N}\log\left[1+\exp\left\{-y_{n}\beta^{⊤}\phi \left(x_{n}\right)\right\}\right]+\sum_{i=1}^{M}{∥w_{i}^{k}\cdot \beta ∥}_{\ell_{2}}. \end{array}$$ (21)

Let us use the following notation:

$$\begin{array}{ll} \alpha^{k+1} & ≜\nabla_{\gamma }v{\left(\gamma ,H\left(\beta^{k+1}\right)\right)}^{⊤}{|}_{\gamma =\gamma^{k}}\\ & =\nabla_{\gamma }{\left(\text{log}\left|\Gamma \right|+\text{log}\left|H\left(\beta^{k+1}\right)\right|\right)}^{⊤}{|}_{\gamma =\gamma^{k}}\\ & =\left[\begin{array}{ccc} \alpha_{1}^{k+1}, & \cdots & ,\alpha_{N}^{k+1} \end{array}\right]. \end{array}$$ (22)

According to the matrix derivative rule, we can derive the expression of the *i*th element of ***α***^*k*^ as:

$$\begin{array}{c} \alpha_{i}^{k+1}=-\frac{H{\left(\beta^{k+1}\right)}_{i,i}^{-1}}{{\left(\gamma_{i}^{k}\right)}^{2}}+\frac{1}{\gamma_{i}^{k}}. \end{array}$$ (23)

According to Eq (19), optimal $\gamma_{i}^{k+1}$ is obtained by minimising

$$\begin{array}{c} \frac{{\left(\beta_{i}^{k+1}\right)}^{2}}{\gamma_{i}}+\alpha_{i}^{k+1}\gamma_{i}. \end{array}$$ (24)

Since

$$\begin{array}{c} \frac{{\left(\beta_{i}^{k+1}\right)}^{2}}{\gamma_{i}}+\alpha_{i}^{k+1}\gamma_{i}\geq 2|\sqrt{\alpha_{i}^{k+1}}\cdot \beta_{i}^{k+1}|, \end{array}$$ (25)

the optimal ***γ*** can be obtained as:

$$\begin{array}{c} \gamma_{i}^{k+1}=\frac{|\beta_{i}^{k+1}|}{\sqrt{\alpha_{i}^{k+1}}},\forall i. \end{array}$$ (26)

The pseudo code is summarised in Algorithm 1.

**Algorithm 1** Reweighted *ℓ*_2_ type algorithm

1: Initialise the unknown hyperparameter ***γ***^1^ as a unit vector;

2: Initialise $w_{i}^{1}=1,\;\forall i$;

3: **for** *k* = 1, …, *k*_max_ **do**

4:

$$\begin{array}{c} \beta^{k+1}=\underset{\beta }{\mathrm{argmin}}\sum_{n=1}^{N}\log\left[1+\exp\left\{-y_{n}\beta^{⊤}\phi \left(x_{n}\right)\right\}\right]+\sum_{i=1}^{M}{∥w_{i}^{k}\cdot \beta_{i}∥}_{\ell_{2}}; \end{array}$$ (27)

5:  $\alpha_{i}^{k+1}=-\frac{H{\left(\beta^{k+1}\right)}_{i,i}^{-1}}{{\left(\gamma_{i}^{k}\right)}^{2}}+\frac{1}{\gamma_{i}^{k}}$;

6:  $\gamma_{i}^{k+1}=\frac{\left|\beta_{i}^{k+1}\right|}{\sqrt{\alpha_{i}^{k+1}}}$;

7:  $w_{i}^{k+1}=\frac{1}{\sqrt{\gamma_{i}^{k+1}}}$;

8:  **if** a stopping criterion is satisfied **then**

9:   Break.

10:  **end if**

11: **end for**

The cost function defined in Eq (27) of Algorithm 1 is convex, making it possible to apply standard solvers to obtain a global optimal solution. For example, we can consider iterative solvers, such as the standard gradient method, the Newton method and its variants. We should note that in the 5^th^ line of Algorithm 1, the inverse of Hessian matrix needs to be calculated. When the number of features is quite large, which is usually the case in the gene expression data, it is quite computational expensive to do the Hessian matrix inversion. Therefore, we apply a Quasi-Newton method, limited memory Broyden Fletcher Goldfarb Shanno (L-BFGS) algorithm, to directly generate the inverse of Hessian matrix in the iterative solver without performing matrix inversion [40].

## Results

In this section, a set of simulated datasets are first used to show the ability of selecting relevant features as well as constructing predictive models. Our method is compared with the other two representative methods: ℓ_1_ regularised logistic regression implemented by alternating direction method of multipliers (ADMM) [41] (code is downloaded from https://web.stanford.edu/~boyd/papers/admm/logreg-l1/) and SBL (code is downloaded from http://www.relevancevector.com). The results will highlight the performance of our method on datasets with high correlations. To demonstrate the applicability of our method on real datasets, we choose a publicly available gene expression dataset for illustration. The identified features (genes) are fed into downstream analyses. We find that the detected functional terms agree with the findings from literature. The analyses of real data show that our method can generate a list of predictive genes that are both used for classifier construction and biological functionality discovery.

### Simulated data analysis

In the simulation, we first generate a dataset with the sample and feature size of 500 and 50. We assume there are only 8 non-zero elements in ***β***. To check the ability of finding correlated features, we split the features with non-zero weights into two sets, *S*^1^ and *S*^2^, each of which contains 4 true features. Then, we initialise a design matrix **Φ** = **x** with 500 samples and 50 features from the normal distribution. Let *ϕ*_*i*_(**x**) be the *i*th column of **Φ**. We set $\phi_{S_{m}^{2}}\left(x\right)=\phi_{S_{m}^{1}}\left(x\right)+N\left(0,0.1\right)$, where $S_{m}^{1}$ and $S_{m}^{2}$ denote the *m*th element in each feature set. In this way, we get data generated from correlated features. The target variable vector ${\left\{y_{n}\right\}}_{n=1}^{500}$ is generated by the linear model using ***β*** and **Φ** with additive independent identical distributed Gaussian noises, where the standard deviation of noise varies ranging from {0, 0.1, 0.5, 1}.

**Fig 1:**
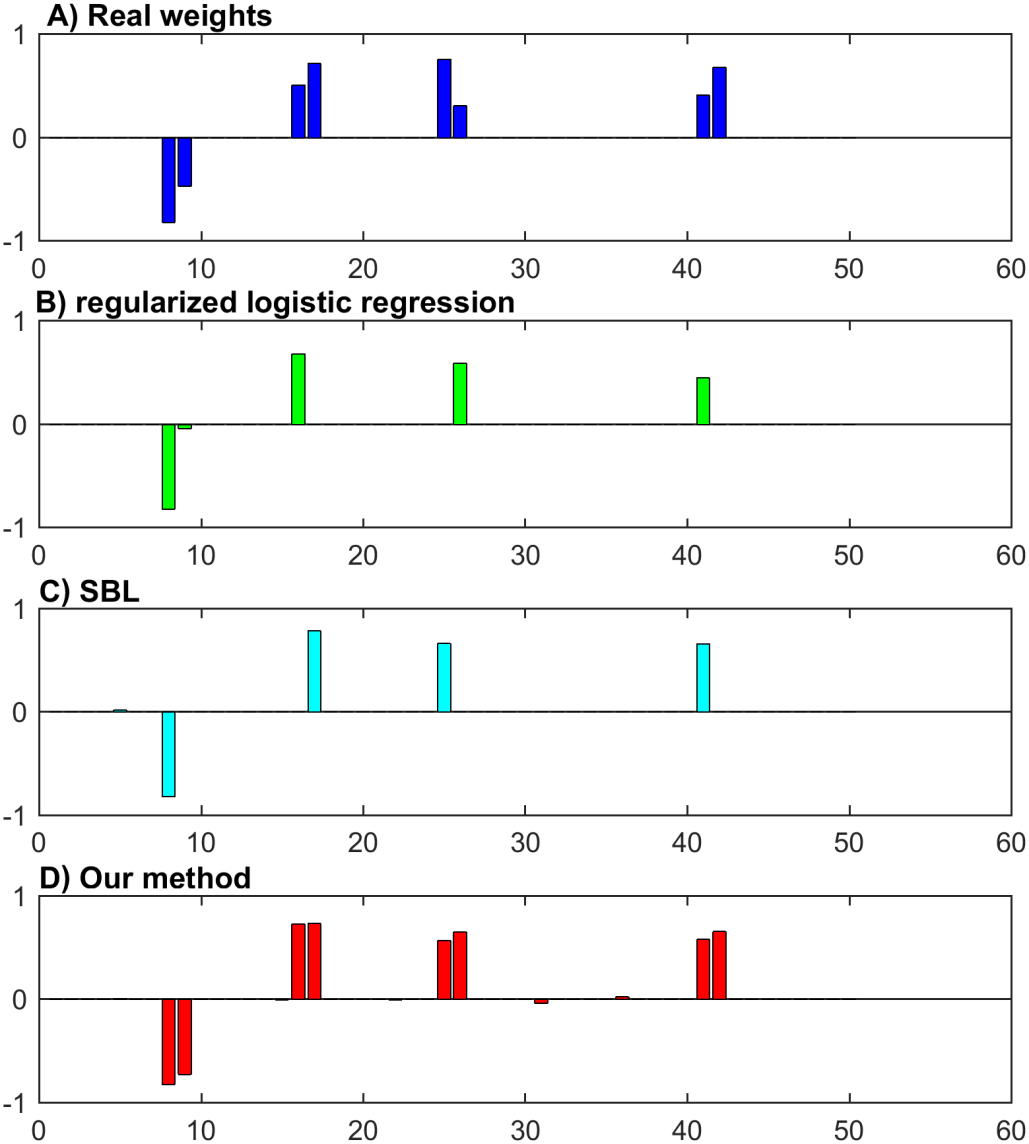
The comparison of the real weights and the weights estimated from different methods.

We first work on the whole dataset of 500 samples with no additive noise to investigate the ability of selecting the true features. The estimated weights from our method is compared with the true weights, the estimates from the ℓ_1_ regularised logistic regression method (RLR) [41] and the sparse Bayesian learning method. We scale the estimated results to make the first real nonzero feature have the same value. The results are shown in Fig 1. It shows that only our method can successfully detect all the true features with magnitudes of the corresponding weights significantly larger than the others. RLR and SBL easily ignore some features that one out of a correlated pair can be detected. This observation is quite encouraging, showing that our method can be potentially used as a good tool to select relevant features. Most classification methods are not guaranteed to have this characteristic.

We then investigate the performance of our method with different levels of noise. The performance is evaluated by the 10-fold cross validation. In the cross validation process, different training dataset is used for feature selection and predictive model construction in each fold. Therefore, selected feature sets from all folds may vary due to the variation of training datasets. To select a feature set which is stable with small fluctuations of the input dataset and also has good predictive accuracy, we use the method from [42]. In the *k*th fold of cross validation, the whole dataset **D** is split into two subsets: CV training dataset **D**_**k**_ and CV testing dataset **D**_\**k**_. Our method can work as a feature selection method on the training dataset **D**_**k**_ to rank and select the top q features, labelled as **V**_**q**,**k**_. After features have been selected, our method then constructs a predictive model for classification using **V**_**q**,**k**_. The prediction results at this CV fold are recorded for later evaluation. To get the complete prediction results, we repeat the above steps for all folds of CV. The method presented in [42] returns an optimal feature set $\tilde{V_{q}}$ with an associated performance score $\tilde{P_{q}}$ under each value of *q*. The score $\tilde{P_{q}}$ is calculated according to the 6^th^ strategy proposed in [43] to assess the prediction accuracy and stability of features (the details of calculating this score can be found in [43]). By checking the maximum value of $\tilde{P_{q}}$, we can determine the optimal value of *q* and the corresponding optimal feature set $\tilde{V_{q}}$. The detected optimal set can then be used to construct a predictive model for future prediction.

**Fig 2:**
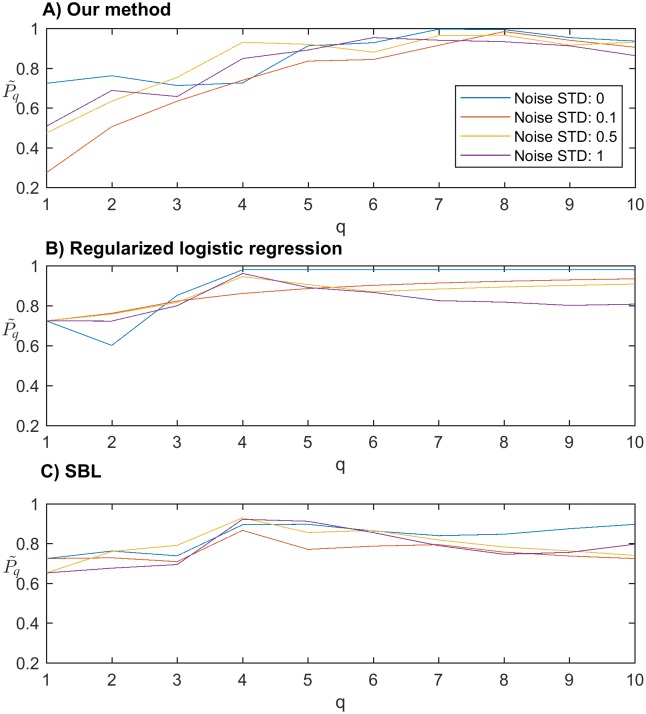
The scores achieved by different methods with the size of feature set varying from 1 to 10 and standard deviation of noise chosen from 0, 0.1, 0.5 and 1.

In Fig 2, it shows the change of scores $\tilde{P_{q}}$ under different settings of feature size *q* and noise level. From Fig 2, we can see that the scores of our method under different noise levels peak when the value of *q* is close to the number of real nonzero features. In contrast to our method, the optimal value of q for the RLR method and the SBL method are around 4, which is different from the real number of nonzero features. From Fig 2, we can expect that our method works better than the RLR method with respect to feature selection. Table 1 further confirms our observations by showing the accuracy values of classification through the 10-fold cross validation. The feature set size *q* under different conditions is chosen to be the optimal value detected from Fig 2. To show the ability of detecting real positives (i.e. real nonzero features), we also present the results of false positive (FP) and false negative (FN) rates in Table 1. We can see that under different noise levels, the accuracy achieved by different methods are all maintained at high levels. However, the false negative rates from other methods are much higher than the rates from our methods. This is because, the other methods cannot detect correlated features that some real features are ignored.

**Table 1:** The results of our method, the regularised logistic regression (RLR) and sparse Bayesian learning (SBL) under different noise levels. The accuracy of classification, false positive and false negative rates are compared.

Noise STD	Method	Accuracy	False Positive	False Negative
**0**	Our method	0.996	0	0
	RLR	0.982	0	0.5
	SBL	0.998	0.024	0.5
**0.1**	Our method	0.986	0	0
	RLR	0.986	0.143	0.5
	SBL	0.992	0	0.5
**0.5**	Our method	0.98	0	0
	RLR	0.972	0	0.5
	SBL	0.98	0	0.5
**1**	Our method	0.972	0.024	0.125
	RLR	0.964	0	0.5
	SBL	0.972	0	0.5

The above observations are only for datasets with fixed sample and feature sizes (500 and 50 respectively). To test generality, we generate a set of datasets as follows: the sample and feature sizes are chosen from {50, 100, 500}; the ratio of non-zero features (sparsity) is either 0.1 or 0.2; non-zero features can be fully independent or 50% of them are highly correlated. For each combination of settings, 20 randomly sampled datasets are generated. We apply our method and also the other two methods on these datasets. The FN, FP and accuracy values are derived from the average values of 20 randomly sampled datasets under each specific setting. The results for the datasets with the sparsity of 10% are recorded in Table 2. The results for the datasets with the sparsity of 20% can be found in S1 Table.

**Table 2:** The performance of our method, the RLR method and the SBL method with the sparsity of 0.1.

%cor	# Sample	# Feature	Method	Accuracy	False Positive	False Negative
0	50	50	Our	0.84	0.0156	0.17
			RLR	0.862	0.0189	0.17
			SBL	0.874	0.0211	0.12
		100	Our	0.686	0.111	0.395
			RLR	0.704	0.108	0.425
			SBL	0.656	0.179	0.61
		500	Our	0.534	0.158	0.746
			RLR	0.52	0.211	0.896
			SBL	0.498	0.219	0.974
	100	50	Our	0.972	0	0
			RLR	0.953	0	0
			SBL	0.97	0	0
		100	Our	0.809	0.0267	0.14
			RLR	0.812	0.025	0.125
			SBL	0.859	0.0233	0.035
		500	Our	0.596	0.139	0.627
			RLR	0.586	0.1941	0.747
			SBL	0.567	0.21	0.892
	500	50	Our	0.993	0	0
			RLR	0.983	0	0
			SBL	0.995	0	0
		100	Our	0.988	0	0
			RLR	0.971	0	0
			SBL	0.986	0	0
		500	Our	0.791	0.0357	0.121
			RLR	0.803	0.021	0.139
			SBL	0.811	0.0666	0.049
50	50	50	Our	0.952	0.0022	0.04
			RLR	0.934	0.0189	0.42
			SBL	0.962	0.0844	0.39
		100	Our	0.811	0.0578	0.12
			RLR	0.794	0.0794	0.515
			SBL	0.809	0.1689	0.52
		500	Our	0.549	0.148	0.633
			RLR	0.545	0.214	0.927
			SBL	0.556	0.219	0.974
	100	50	Our	0.985	0	0
			RLR	0.964	0.0011	0.39
			SBL	0.987	0.0622	0.4
		100	Our	0.959	0	0
			RLR	0.952	0.0017	0.49
			SBL	0.969	0.0894	0.48
		500	Our	0.634	0.139	0.45
			RLR	0.631	0.199	0.796
			SBL	0.592	0.2113	0.902
	500	50	Our	0.995	0	0
			RLR	0.982	0.106	0.37
			SBL	0.995	0.0378	0.39
		100	Our	0.995	0	0
			RLR	0.98	0.0628	0.465
			SBL	0.992	0.131	0.48
		500	Our	0.938	0.0026	0.023
			RLR	0.931	0.00011	0.501
			SBL	0.592	0.145	0.455

We can see that for the datasets with independent features, the performance of our method is similar with the other two methods. However, for the datasets with correlated features, our method works much better than the other two methods with lower FN and FP rates and similar accuracy values. Especially, the FN rates are much smaller, indicating that the real non-zero features are more likely to be detected by our method. This characteristic of our method is quite important, since biological expression data contains a lot of correlated features. We can also observe a general trend that: with a fixed sample size, the performance of all three methods is reduced when the feature size increases; with a fixed feature size, the performance of all three methods can be improved with more samples. When the proportion of non-zero feature increases to 0.2 (see S1 Table), the performance of all methods are deteriorated. This is because, all these methods are based on the assumption that the feature space is sparse. For biological expression data, the ratio of predictive features or biomarkers is quite low, which is much smaller than 0.2 or even 0.1.

### Embryonal tumour gene expression data analysis

We use a public available gene expression dataset of the central nervous system embryonal tumours from the study in [8]. All relevant data are available from the figshare repository at the following URL: https://doi.org/10.6084/m9.figshare.5678806.v1. We selected 10 CNS medulloblastomas (MD) samples and 10 non-neuronal origin malignant gliomas (Mglio) samples to show the performance of our method in classifying two tumour types. The samples were hybridised on Affymetrix HuGeneFL GeneChip arrays. We first preprocessed the raw data using GCRMA with empirical Bayes estimate [44]. Then we filtered out probe sets which are either not annotated or have little variability across samples. Probes for 5669 genes were remained after preprocessing.

**Fig 3:**
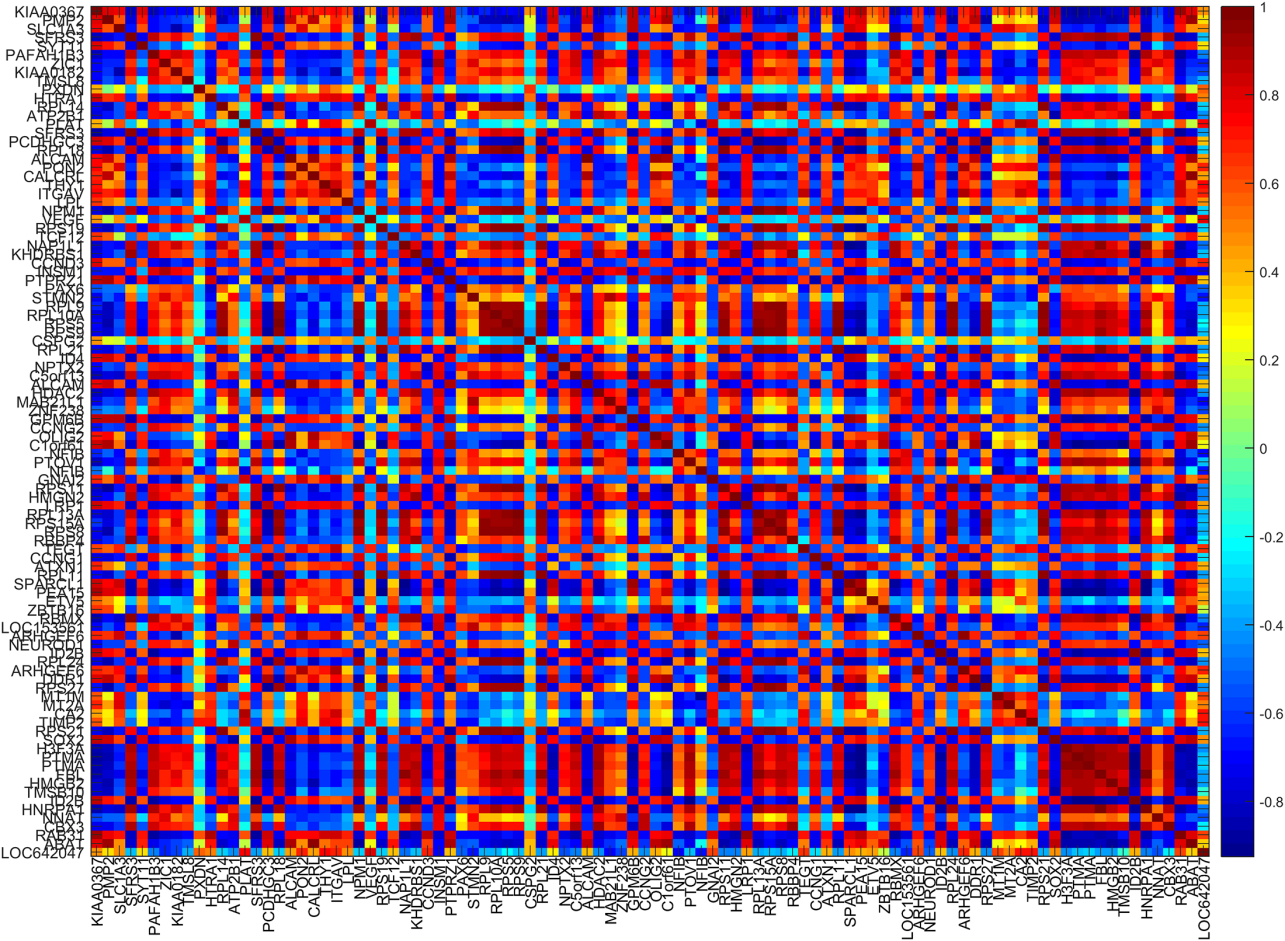
Heatmap of the correlation matrix for 98 genes selected by our classifier.

Our method can find differences between two tumour types at molecular level. We construct a classifier using the selected 20 samples with the accuracy of tumour type prediction approaching to 100% in the 10-fold cross validation procedure. The beauty of our method is that it does not only have strong predictive power, but also selects relevant features that could be candidates of disease biomarkers. The following parts of this section focus on investigating the performance of features selection. In the classification model of the whole dataset, 98 features have non-zero weights, which can be regarded as molecular features distinguishing tumours. By looking at Fig 3, we can see that many of these features are highly correlated, telling that our method does not discard features from correlated ones. We also apply SBL to construct a classification model. Although it can also return high predictive accuracy, it only selects two features with non-zero weights in the model. As RLR cannot work well for large datasets, we do not present its results for comparison.

**Fig 4:**
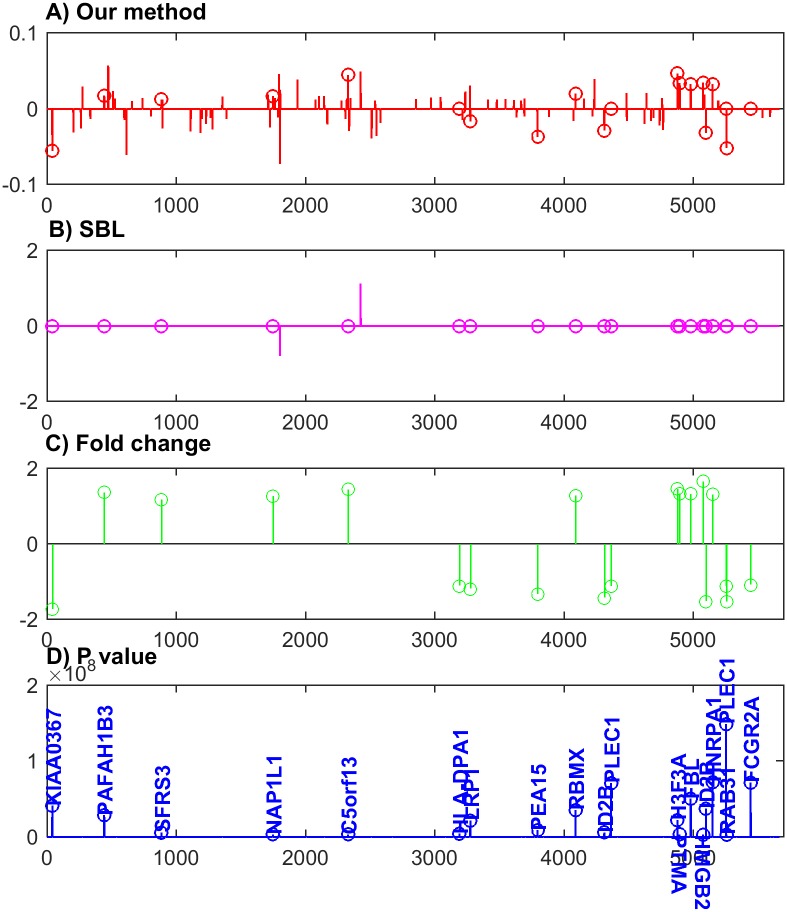
The comparisons for the estimated weights from our method and SBL with fold changes and *p* value. The top 20 features from *t* tests are shown as circle points. A) and B) are weights of features from the classification models using our method and SBL, respectively; C) shows fold changes of the top 20 statistically significant features; D) shows the exp(−log(⋅)) transformed *p* values for the top 20 statistically significant genes (labelled by official gene symbol).

The weights of features estimated from our method are compared with significant levels of features from the traditional statistics tests, which check features one by one to see whether the distributions of each feature in different groups are significantly different. Only the features whose p-values are smaller than the significant level (e.g., 0.05) are selected. Although there are many *p* value correction methods, a hard cut-off value is still needed. The number of selected features depends on the value of the significant level. Although it is conventional to set the significant level to be 0.05 or 0.01, we can hardly say any features whose p-value is slightly larger than this value do not have discriminant power. In this experiment, a t-test for each gene is conducted to find significant changes in expression levels between the MD and Mglio samples. We show the top 20 genes with the smallest *p* values in Fig 4.

Fig 4 compares the weights resulting from our method and SBL with fold changes and *p* values. Fig 4A) shows the weights of features in our classification model, where the circle points indicates the top 20 features from the *t* test. We can see that most features which are statistically significant have non-zero weights. In contrast to our method, Fig 4B) shows the results from SBL, telling that the top 20 features from the *t* test all have zero weights. From Fig 4A) and 4B) we can see our method outperforms SBL in the aspect of feature selection. Fig 4C) shows the fold change of the top 20 statistically significant features. Comparing Fig 4A) with 4C) we can see that the signs of weights agree with the signs of fold changes. It demonstrates that our method can reflect whether a gene is up-regulated or down-regulated for MD compared to Mglio. Fig 4D) shows the exp(−log(⋅)) transformed *p* values for the top 20 statistically significant genes. It should be noted that although we compare our results with *p* value results, we cannot simply regard *p* value results as the ground truth. The disagreements between our method and *t* test can be resulted from the case that *t* test gives wrong results. This is because, in the statistic tests, features are investigated separately. It is often the case that some individual features are not discriminant but have strong predictive power when they join together. If we compare our results with the ground truth, we may find a better comparison results than those shown in Fig 4. As the ground truth is not at hand, we use *p* value results for comparison.

**Fig 5:**
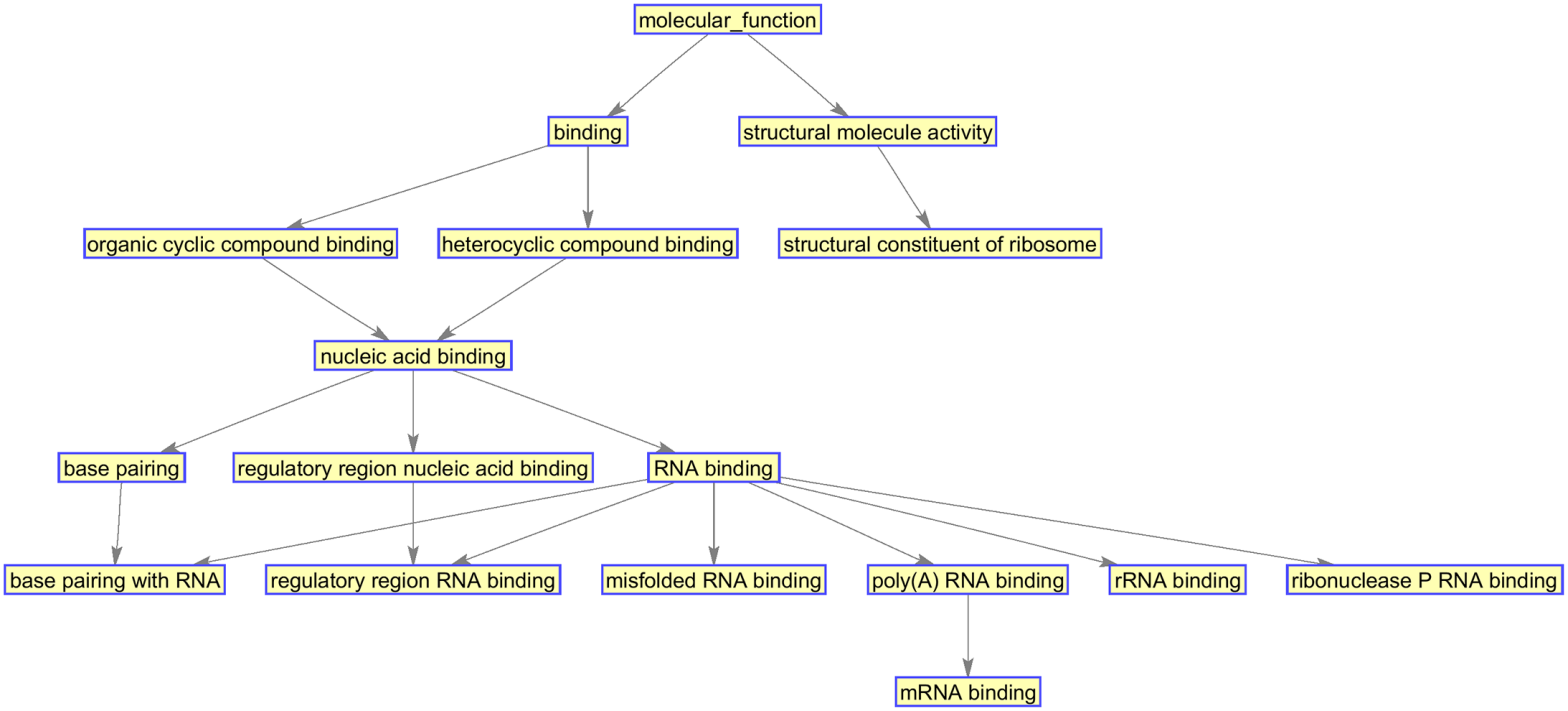
The ontology built using the top 10 significant GO terms discovered from up-regulated genes for MD compared to Mglio.

After comparing our results with other methods, we then investigate the biological functions of the 98 selected genes from our method. In this experiment, we determine a list of 53 up-regulated genes for MD compared to Mglio. Genes from this list are analysed for functional category enrichment using the Functional Annotation Clustering tool on the Database for Annotation, Visualisation and Integrated Discovery [45]. Metastasis-associated genes are classified according to their annotated role in molecular function, biological process, and cellular component from Gene Ontology (GO). Category enrichment is tested against all human genes, where *p* values are adjusted using the Benjamini-Hochberg multiple testing correction method [46]. In S2 Table, we show the discovered GO annotation clusters, where the detected GO terms are consistent with the findings in [8]. We can further select the GO terms of interest and build a sub-ontology that includes the ancestors of the terms. Fig 5 shows the ontology built using the top 10 significant GO terms. From this experiment, we can see that our method can work as a classifier and also a feature selection method whose output (selected features) can be fed into downstream analyses such as gene set enrichment and pathway analysis.

## Discussion

With the development of high-throughput technology, biological process can be quantitatively measured. Differential feature detection and classification model construction are two main analyses in the biological expression study. In this paper, we propose a method to perform these two analyses at the same time: the model can predict sample groups; and the features used in the model with non-zero weights can be regarded as potential biomarkers. Currently, there are many machine learning methods for classification model construction. Most of them *cannot* directly return a list of predictive features with non-zero weights in the model. For example, the linear SVM may use all features for model construction, where they all have non-zero weights.

As SBL imposes sparsity to the model, a lot of features are forced to be pruned in the classification model. Thus, we use SBL as the basis of our method. Different from SBL which follows an EM style to infer parameters and hyperparameters iteratively, we formulate the inference problem in the framework of optimisation: the target function in the optimisation process is originated from SBL; the iterative updating procedure follows the idea of convex-concave optimisation. Compared with SVM-based methods, our method has the following distinguishing features: 1) Our method is parameter free that hyperparameters are directly learned from datasets, while SVM-based methods need to set parameters through the cross validation process. 2) Our method imposes sparse constraints to the classification model. By choosing linear kernel, we can obtain a small set of features with non-zero weights used in model. The feature selection and classification steps have been integrated into one step. 3) Our method can detect correlated features which are important for downstream analysis, such as functional pathway analysis. Our method is also different from other optimisation based methods. Let us take the BPSO method as an example for discussion. The main differences between our method and BPSO are: 1) The BPSO method is inherently a global optimisation method. Our method although is an optimisation method, it constructs the model from the Bayesian point of view, where prior knowledge can be explicitly included in the model. 2) Our method is parameter free that hyperparameters are learned from the data. BPSO needs to set parameters in advance or obtained them via the cross validation process. 3) Looking at the results from BPSO, we can see that in each run of BPSO, only a small subset of genes is selected (e.g., 10 genes). 4) Our method carries out classification and feature selection in one step, where BPSO is mainly used for feature selection requiring other classification method such as ELM for classification model construction.

The simulation results show that our method can effectively select features with high classification accuracy. In contrast to other methods, correlated features can be successfully detected. A real gene expression data from the embryonal brain tumour study is then used to demonstrate the applicability of our method. In the results, we first show that the selected features are correlated by looking at the heatmap of correlation matrix. Then we compare the weights estimated from our method with *p* values from statistic test and fold changes. We find that our method can successfully identify up-regulated and down-regulated genes with positive and negative weights, respectively. Moreover, we find that most features which are statistically significant have non-zero weights in our model. The gene list generated by our method can be used to do functional analysis. We show the detected gene ontology terms, which are consistent with the findings in previous study. In conclusion, the classification and feature selection method proposed in this paper can effectively handle highly correlated biological expression dataset, in order to predict distinct disease subtypes and select candidates of biomarkers simultaneously.

## Supporting information

S1 Table The performance of our method, the regularized logistic regression method (RLR) and the SBL method with the sparsity of 0.2. The number of samples and features is chosen from 50, 100, 500. The percentage of correlated features (%cor) can be 0 or 50.
(XLS)

S2 Table The discovered GO annotation clusters. (XLS)
